# Feeling more than understanding: empathic disequilibrium and emotional reactivity in eating psychopathology

**DOI:** 10.1186/s40337-026-01600-2

**Published:** 2026-04-18

**Authors:** Laura Vuillier, Ido Shalev, Rachel Louise Moseley, Florina Uzefovsky

**Affiliations:** 1 Department of Psychology, Bournemouth University, Poole, UK; 2 Medical Research Council Cognition and Brain Sciences Unit, University of Cambridge, Cambridge, UK; 3 Department of Psychology, Ben-Gurion University of the Negev, Beersheba, Israel; 4 The Duet Center, Ben-Gurion University of the Negev, Beersheba, Israel

## Abstract

**Background:**

Emotional dysregulation is a core feature of eating disorders, yet research has predominantly focused on *intra*personal emotion processes rather than *inter*personal emotional mechanisms. Empathy comprises affective empathy (AE; *feeling* others’ emotions) and cognitive empathy (CE; *understanding* others’ emotions), with recent research suggesting that empathic disequilibrium—imbalances between AE and CE—may contribute to psychopathology. We hypothesized that empathic disequilibrium characterized by AE-dominance underlies emotional difficulties in eating disorders through heightened emotional reactivity.

**Methods:**

We conducted a two-phase investigation. Study 1 examined empathy and eating disorder symptoms in 345 undergraduate students using the Interpersonal Reactivity Index (IRI) and Eating Disorder Examination Questionnaire (EDE-Q). Study 2 replicated findings in 835 participants (including 103 with eating disorder diagnoses) and tested emotional reactivity as a mediator using the Emotional Reactivity Scale (ERS).

**Results:**

Both studies demonstrated consistent associations between empathic disequilibrium characterized by AE-dominance and eating disorder pathology (Study 1) and diagnosis (Study 2), with CE being unrelated to eating disorder symptoms. Mediation analyses revealed that emotional reactivity mediated the relationship between empathic disequilibrium and eating disorder symptoms, with sensitivity analyses supporting pathway robustness.

**Conclusions:**

This study provides first comprehensive evidence that empathic disequilibrium, rather than specific empathic deficits, represents a potential risk factor for eating psychopathology. AE-dominance appears to create emotional hyper-arousal when encountering others’ emotions, which may be regulated using disordered eating behaviours. These findings challenge traditional empathy approaches in psychopathology and highlight the importance of interpersonal emotional processes in eating disorder conceptualization and treatment, opening new therapeutic avenues targeting both intrapersonal and interpersonal emotional functioning.

**Supplementary Information:**

The online version contains supplementary material available at 10.1186/s40337-026-01600-2.

## Abstract (plain language summary)

People with eating disorders often struggle with managing emotions, but most research has focused on how they handle their own emotions rather than how they respond to other people’s emotions. This study examined empathy imbalance: when someone is much better at *feeling* others’ emotions than *understanding* them, or vice versa. We studied over 1000 people across two studies, including over 100 individuals with eating disorders. We found that people with eating disorders tend to have an empathy imbalance where they *feel* others’ emotions very intensely but struggle to *understand* them cognitively. Our findings suggest that this imbalance makes them emotionally overwhelmed when around other people’s emotions, potentially leading them to use eating disorder behaviours to cope with these intense feelings. We suggest that helping people with eating disorders balance their empathy, such as by learning to understand emotions without becoming overwhelmed by feeling them, could be an important new treatment approach alongside current therapies.

**Supplementary Information:**

The online version contains supplementary material available at 10.1186/s40337-026-01600-2.

## Background

Whilst emotional dysregulation represents a core feature of eating disorders [1–3], research has so far mainly focused on difficulties regulating emotions in general, rather than focusing on our responses to others’ emotions more specifically. This emphasis on internal emotional mechanisms, while valuable, indeed represents only one dimension of emotional functioning [4], and less attention has been devoted to interpersonal emotion processes in eating disorders, particularly the ways in which individuals understand and respond to the emotions of others, through empathy (but see [5, 6]), despite social and emotional difficulties being known key factors in the development and maintenance of the illness (e.g. [7, 8]). Empathy, broadly defined as the ability to understand and share the emotional experiences of others while maintaining a self-other distinction ([9, 10], comprises two distinct but interrelated components: affective empathy (AE: the ability to *feel or share* someone else’s emotions) and cognitive empathy (CE: the ability to *understand* others’ emotional states). While these components are typically studied independently (e.g. [6]), they are suggested to work in tandem to regulate interpersonal emotional responses [11]. In typical development, adults exhibit a state of empathic equilibrium wherein their AE and CE are essentially proportionate to one another in strength or proficiency [12, 13]. However, emerging evidence indicates that this balance may be disrupted in certain conditions. Recent research suggests that imbalances or disequilibrium between AE and CE, irrespective of overall levels of empathy, may contribute to interpersonal challenges and psychopathological processes of different kinds in autism, schizophrenia, as well as in individuals with psychopathic tendencies or symptoms of depression and anxiety [13–17]. Such ‘empathic disequilibrium’—characterised by disparities between one’s capacity to feel versus understand others’ emotions—has not yet been studied in eating disorders. However, given the well-established co-occurrence of autism and eating disorders [18–22]—in part linked to shared emotional difficulties [23–26]—and the presence of empathic disequilibrium in autistic people and individuals with high autistic traits [16, 17, 27, 28], it is plausible that individuals with eating disorders may exhibit a similar empathic profile. The present study proposes that empathic disequilibrium may serve as a previously underexplored mechanism underlying the social and emotional difficulties experienced by people with eating disorders, potentially offering new avenues for conceptualisation as well as treatment and support.

Emotional difficulties manifest across multiple domains in individuals with eating disorders, including difficulties with emotion regulation [1, 2, 29], alexithymia—the inability to identify and describe one’s own emotions [30], as well as maladaptive beliefs about the controllability or usefulness of emotions [31, 32]. Another factor implicated in emotional difficulties in eating disorders is emotional reactivity, which refers to the intensity, speed, and duration of emotional responses [33]. Research has reliably shown that people with eating disorders exhibit heightened emotional reactivity, characterized by intense emotional responses [34, 35]. This heightened emotional reactivity is thought to overwhelm an individuals’ capacity for adaptive emotion regulation, leading to the adoption of maladaptive coping strategies such as disordered eating behaviours (binge eating, purging, or restriction) in eating disorders. However, whilst most research has tried to understand the role of emotional reactivity through an intrapersonal lens (i.e. the emotional reaction to an intrapersonal situation such as feeling distressed after making a mistake at work), it remains to be explored whether the process by which other people’s emotions affect our own is also involved (i.e. an intrapersonal process within an *inter*personal context, such as feeling distressed about someone else’s distress). As such, we hypothesise that an imbalance between the ability to *feel* and *understand* the emotions of other people could explain the heightened emotional reactivity seen in eating disorders.

The literature on empathy in eating disorders reveals distinct patterns of empathic functioning that vary by empathy and eating disorder type. Research has predominantly focused on anorexia nervosa (AN), with meta-analytic evidence indicating that individuals with AN show significantly lower CE compared to healthy controls, while AE scores do not differ [6]. More recent work corroborates lower CE in people with AN, whilst finding the status of AE more uncertain [5, 36], and suggests differences might be more stable than state-dependent [5] and less apparent if empathy is examined as a whole [37]. Only three studies have explored empathy in bulimia nervosa (BN) and binge eating disorder (BED), and only one looked at CE and AE separately, showing that higher-weight women with BED scored higher on the personal distress scale (a component of AE) than matched comparison women without BED [38]. Others, in line with transdiagnostic approaches to eating disorders, collated participants with different diagnoses: while some observed no group differences in CE and AE in direct contrasts between eating disorder groups and healthy controls [39], others observed significant relationships between shared eating psychopathology and difficulties in understanding and sharing other people’s emotional states [40]. These inconsistent findings suggest that empathic functioning may be more complicated than originally thought, and it is possible that looking at the balance—or imbalance—between CE and AE may be more relevant to explain social difficulties and psychological distress seen in eating disorders rather than looking at these concepts separately.

While both CE and AE are important for effective social and psychological functioning, recent research has begun to explore the concept of empathic disequilibrium—the imbalance between cognitive and affective empathy within an individual. This concept has gained traction with regards to explaining empathic interpersonal differences in autistic people [16, 27, 28], individuals who self-harm [16]—a behaviour with similar links to difficulties with emotion processes in eating disorders [41, 42]—as well as symptoms of anxiety and depression in the general population [15]. In their 2023 paper, Shalev et al. [15] for example showed that the relative *imbalance* of empathic processes, towards either extreme, explained unique variance in the symptoms of anxiety and depression, with higher affective relative to cognitive empathy (AE-dominance) linked to anxiety symptoms. They also showed that the relationships between AE-dominant empathic disequilibrium and anxiety were mediated by emotional reactivity [15], which was also seen for non-suicidal self-injury (NSSI) in autistic people [16]. Conceptually, equilibrium between empathic processes ensures that empathic responses are appropriately and affectively up- or down-regulated [11, 43]. Instances of AE-dominance would reflect insufficient down-regulation of emotional empathic responses by cognitive empathic processes, resulting in greater reactivity and hyper-arousal in response to others’ emotions. This example demonstrates that even in instances where AE and CE appear relatively ‘intact’ in relation to total scores, the magnitude of discrepancy between them, in and of itself, could explain unique variance in interpersonal difficulties and related psychopathology in eating disorders.

In that eating disorders resemble anxiety and indeed NSSI with regards to intrapersonal differences in emotion processes, we might expect individuals with eating disorders to show a pattern of AE-dominance (as might be suggested by difficulties with CE). If this AE-dominance pattern exists, it could create a relationship whereby AE-dominance leads to emotional hyper-arousal (as reflected by emotional reactivity), which would lead to disordered eating behaviours, given their function as a means of emotion regulation. However, the inconsistency of previous empathy research in eating disorders leaves queries regarding the status of both empathic processes and their relationship to each other. While AE and CE have been examined individually in eating disorders and yielded mixed findings, the question as to whether empathic disequilibrium towards either CE- or AE-dominance explains unique variance in eating psychopathology, over and above contributions from overall empathy, is yet to be addressed.

## The current study

Given the inconclusive nature of the existing literature on empathy in eating disorders, and the potential of empathic disequilibrium as a unique contributor to emotional reactivity and eating psychopathology, the present study comprised a two-phase investigation. In the first phase (Study 1), we collected data in *N* = 345 participants, assessing their levels of CE and AE (using the Interpersonal Reactivity Index [IRI] [44]), as well as symptoms of eating disorders (using the Eating Disorder Examination Questionnaire [EDE-Q] [45]). In the second phase (Study 2), we expanded our investigation to a larger sample of *N* = 835 participants (including *N* = 103 with an eating disorder), to replicate our findings and test the mediating role of emotional reactivity [as measured with the Emotional Reactivity Scale (ERS) [46]] in the relationship between empathy and psychopathology. We hypothesised that:

1. Eating psychopathology would be associated with empathic disequilibrium. We expected this imbalance to be towards more AE. This was tested in Study 1 with replication in Study 2.
2. Eating disorder diagnosis would be associated with empathic disequilibrium towards AE-dominance. This was tested in Study 2.
3. Emotional reactivity would mediate the link between empathic disequilibrium and eating psychopathology symptoms. This was tested in Study 2.

## Study 1

### Methods

#### Participants

A total of 345 undergraduate students from Bournemouth University in the United Kingdom participated in the study. The majority of the sample identified as White (*N* = 295), including White British, White Irish, and White Other. Smaller proportions identified as Black, Black British, or African-Caribbean (*N* = 11), and Asian or Asian British (*N* = 12). Twenty-one participants identified as mixed race (e.g., White and Asian, White and Black, or other combinations), and five participants reported other ethnic backgrounds (Latin-American, Persian, or Turkish). One participant chose not to disclose their ethnic identity. In terms of sex assigned at birth, 70.14% (*N* = 242) reported being assigned female, and 28.41% (*N* = 100) assigned male. Regarding gender, most participants identified as cisgender women (*N* = 229) and cisgender males (*N* = 98). In addition, 5 identified as transgender men, 5 as non-binary, and 3 reported other gender identities (e.g., agender, genderqueer). Two participants assigned male at birth identified as gender fluid or another identity. Three participants did not report their assigned sex and gender. Most participants identified as heterosexual (*N* = 248; 71.88%), while others identified as bisexual (*N* = 63; 18.26%), homosexual (*N* = 19; 5.5%), pansexual (*N* = 6), queer (*N* = 2), asexual (*N* = 2), or reported another sexual orientation (*N* = 3). The mean age of participants was 20.42 years (*SD* = 4.56; range = 18–64).

Participants were asked whether they had ever received a formal eating disorder diagnosis (yes/no) and, if so, to report the specific condition(s) in a text box, provide the year of diagnosis, and current status (‘*My eating disorder is still active*’, ‘*I had thought I was in remission but recently I’ve been having ED thoughts*,* symptoms and behaviours*’, ‘*I consider myself to be in remission (not troubled by eating disorder symptoms)*’ and had the opportunity to write something else freely). Sometimes, moreover, participants indicated multiple ED diagnoses, to which extent it was unclear which were remitted and which still current. As such, for consistency, and given lasting emotional and empathic differences in people with ED, we included individuals who indicated remitted illness within the ED group. Participants were then asked whether they had ever been diagnosed with any other mental health conditions or as neurodivergent. For subsequent analyses, these self-reported diagnoses were used to characterise the sample’s mental health profile and to derive binary indicators of the presence or absence of key diagnostic categories, including eating disorders.

Most participants reported no history of mental health diagnoses (*N* = 237; 68.70%). Of the 93 participants who had received a diagnosis, 66 reported anxiety disorders, with generalised anxiety disorder being the most common (*N* = 13), followed by social anxiety (*N* = 9), and agoraphobia (*N* = 2), while the remaining diagnoses were unspecified. Depression was reported by 52 participants, followed by obsessive-compulsive disorder (OCD; *N* = 9), borderline personality disorder (*N* = 8), post-traumatic stress disorder or complex PTSD (*N* = 9), psychotic spectrum disorder (*N* = 1), and one individual identified gender dysphoria. Forty-one participants reported comorbid conditions, most commonly involving both depression and anxiety (*N* = 24), or a combination of depression, anxiety, and an additional condition (*N* = 12). A small minority identified that they had been diagnosed as neurodivergent (Autism: *N* = 17, ADHD: *N* = 11). Of the overall sample, 16 (4.64%) participants reported a diagnosis of an eating disorder. Due to the study’s focus, they were asked to specify the diagnosis, year of diagnosis, and current status. Seven participants had been diagnosed with anorexia nervosa, five with bulimia nervosa, one with avoidant/restrictive food intake disorder, and one with other specified feeding or eating disorder. The remaining two did not specify. Of those diagnosed, three reported their eating disorder as currently active, two considered themselves in remission, and eleven described being in remission but expressed concern about residual symptoms. All individuals were able to provide the date of diagnosis.

After providing informed consent, participants completed a series of online questionnaires via Qualtrics. This study received ethical approval from the Research Ethics Panel at Bournemouth University in the UK (ID: 45304).

#### Measures

Empathy: Empathy was assessed using the Interpersonal Reactivity Index (IRI [44]), a self-report instrument consisting of 28 items rated on a 5-point Likert scale. While there is no gold standard for measuring empathy [47], the IRI is the most commonly used measure, which has been correlated with other empathy and neural empathy-related measures [47–49]. The scale includes four subscales, two of which measure cognitive empathy (Perspective Taking and Fantasy), and two which measure affective empathy (Empathic Concern and Personal Distress). Internal consistency was good (McDonald’s ω = 0.79 for cognitive empathy; ω = 0.78 for affective empathy).

Eating disorder symptoms: The Eating Disorder Examination Questionnaire (EDE-Q [45]) was used to assess disordered eating attitudes and behaviours. The 28-item self-report measure uses a 7-point Likert scale and provides subscale and global scores. The scale demonstrates excellent sensitivity and specificity in discriminating between individuals with and without eating disorders. For this study, we used the global score, which showed high internal consistency (McDonald’s ω = 0.96). Different cut-offs have been proposed for the EDE-Q, underscoring its sample specificity [50–53]. However, a global EDE-Q score of *≥* 2.8 is commonly used in research as a clinical threshold balancing sensitivity and specificity in adult samples [50, 51].

In order to identify relationships between disordered eating and empathic processes, we controlled for anxiety and depression, two conditions which commonly co-occur with eating disorders [54].

Anxiety symptoms: Symptoms of anxiety were measured using the Generalised Anxiety Disorder-7 scale (GAD-7 [55]). This 7-item self-report scale demonstrates strong construct and criterion validity and assesses the frequency of anxiety symptoms over the past two weeks using a 4-point Likert scale ranging from “not at all” to “nearly every day.” Higher total scores indicate greater symptom severity. The GAD-7 demonstrated excellent reliability (McDonald’s ω = 0.92).

Depressive symptoms: Depressive symptoms were assessed using the 5-item version of the Center for Epidemiologic Studies Depression Scale (CES-D [56]). Participants rated how often they experienced each symptom in the past week on a 4-point scale from “rarely or none of the time” to “most or all of the time”. This brief version of the CES-D reduces participants’ burden while maintaining good validity. Internal consistency for this scale was acceptable (McDonald’s ω = 0.76).

#### Missing data handling

The overall proportion of missing data across key variables was low (3.04%). Table 1 provides the percentage of missing data by variable. Little’s MCAR test indicated that the data were missing completely at random (*χ*²(6) = 8.22, *p* = 0.22). Missing values were addressed using full information maximum likelihood, incorporating age and unmodelled symptom variables as auxiliary variables via the *semTools* package (v0.5-6.941 [57]).

#### Statistical analysis

Empathic disequilibrium, in essence, is an intrapersonal discrepancy between an individual’s cognitive and affective empathy. However, measuring this discrepancy requires careful methodological consideration, as traditional difference scores (e.g., simply subtracting one from the other) have known limitations in terms of interpretability and psychometric robustness [58, 59]. To address these concerns, we assessed empathic disequilibrium using a Polynomial Regression with Response Surface Analysis, following the approach previously employed by Shalev and colleagues to measure empathic disequilibrium [15, 27]. This method involves conducting a polynomial regression that includes both cognitive and affective empathy, their interaction term, and their respective squared terms. Based on the resulting coefficients, surface parameters are derived corresponding to the additive combination of cognitive and affective empathy (referred to here as overall empathy), and the discrepancy between them (referred to as empathic disequilibrium). For each of these components, both linear effects (e.g., greater overall empathy or cognitive empathy dominance) and nonlinear effects (e.g., curvilinear associations reflecting extreme values or high levels of imbalance) were examined. These relationships are visualised using a surface plot.

Three separate polynomial regression models were conducted to predict eating disorder symptoms. As the raw scores of cognitive and affective empathy (as measured by the IRI) were not intended to be directly compared, potential biases may arise from item wording or scale construction. Therefore, to minimise these biases and ensure comparability between the two scales, both variables were standardised prior to their inclusion in the models. Since prior evidence that empathic disequilibrium may be influenced by sex [15, 27], sex was included as a covariate in all analyses.

In eating disorders, anxiety, and depression symptoms are often interrelated, with high rates of comorbidity [54]. To preserve ecological validity, the primary analyses did not control for co-occurring symptoms. However, we conducted secondary analyses in which anxiety and depressive symptoms were included as covariates to better isolate the unique contribution of empathic disequilibrium to eating disorder symptoms.

All analyses were conducted in R (v4.4.1 [60]), using the *lavaan* package (v0.6-18 [61]) for model estimation. Visualisations of the response surface were generated using the *RSA* package (v0.10.6 [62]).

#### Power analysis

Participants were recruited based on availability through the university’s recruitment system. However, to make sure the sample size was adequate for the planned analysis, we employed a Monte Carlo simulation with 5000 resamples. This analysis estimated the minimum effect size that could be detected with sufficient power, assuming a population structure informed by previous research (e.g. [15]), . The results indicated that our sample size provided adequate statistical power (*1 − β* = 0.82, *α* < 0.05) to detect a small effect size (*r* = 0.21) for the surface parameters. The simulation was conducted using the *simsem* package (v0.5-16 [63]).

### Results

Descriptive statistics are reported in Table 1, and a correlation matrix is shown in Table 2. Individuals who reported having received an eating disorder diagnosis had a mean EDE-Q score of 3.45 (*SD* = 1.45), which was significantly higher than that of individuals without an eating disorder diagnosis (*t*(333) = 3.48, *p* = 0.001, *d* = 0.89).

**Table 1:** Descriptive statistics of Study 1

Variable	Mean (SD)	% Missing data
IRI—cognitive empathy	34.71 (7.97)	2.02
IRI—affective empathy	32.88 (7.52)	2.02
EDE-Q	2.18 (1.52)	2.89
CES-D	6.56 (3.63)	3.48
GAD-7	9.63 (6.45)	3.77

*IRI* Interpersonal Reactivity Index, *EDE-Q* Eating Disorder Examination Questionnaire, *CES-D* Center for Epidemiologic Studies Depression Scale, *GAD-7* Generalised Anxiety Disorder Scale

**Table 2:** Correlation matrix

Variable	1	2	3	4
1. Cognitive empathy				
2. Affective empathy	0.39**			
3. EDE-Q	0.07	0.27**		
4. GAD-7	0.13*	0.32**	0.42**	
5. CES-D	0.12*	0.25**	0.48**	0.67**

*EDE-Q* Eating Disorder Examination Questionnaire, *CES-D* Center for Epidemiologic Studies Depression Scale, *GAD-7* Generalised Anxiety Disorder Scale. * *p* < 0.05. ** *p* < 0.01

**Fig. 1:**
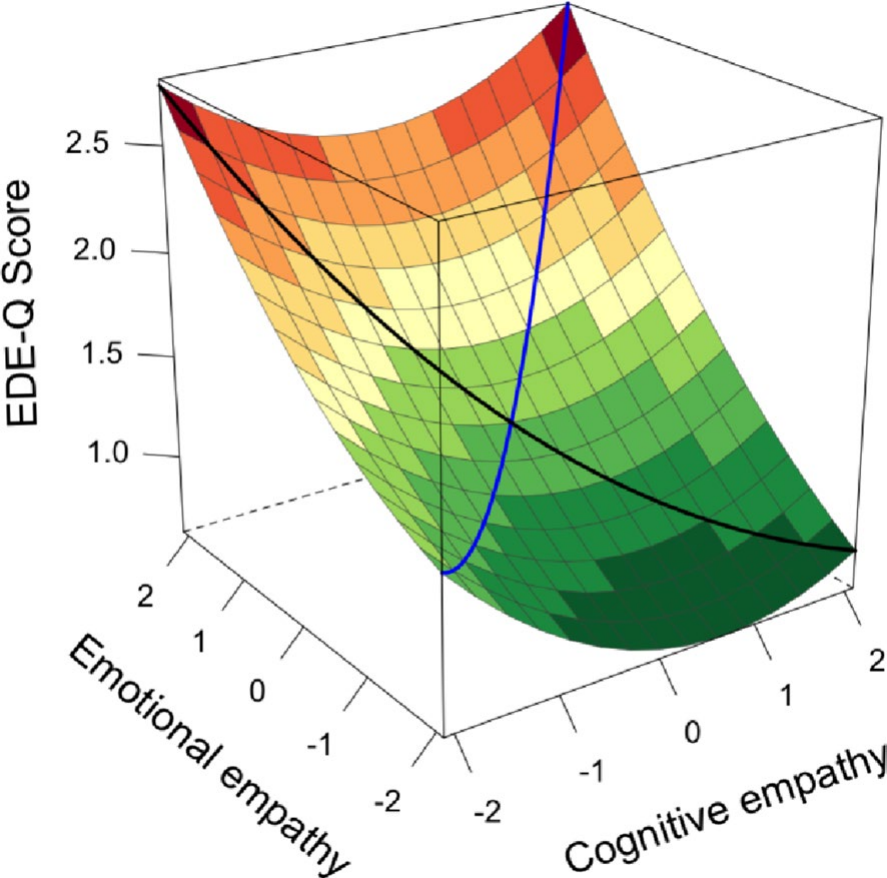
Polynomial regression with response surface analysis predicting eating disorder symptoms. The black line represents empathic disequilibrium, with movement toward the left corner indicating AE dominance and movement toward the right corner indicating CE dominance. The blue line represents overall empathy, with movement along this line reflecting increasing levels of combined AE and CE. Eating disorder symptom severity is represented by the colour gradient, with cooler colours (dark green) indicating lower symptom severity and warmer colours (yellow to red) indicating higher symptom severity

The polynomial regression model predicting eating disorder symptoms explained 10.6% of the variance in EDE-Q scores. As expected, females reported significantly higher levels of eating disorder symptoms than males (*b* = 0.41, 95% CI [0.05, 0.78], *β* = 0.12, *p* = 0.03). As shown in Fig. 1, empathic disequilibrium characterised by AE dominance was significantly related to greater eating disorder severity (*b* = − 0.47, 95% CI [− 0.76, − 0.18], *β* = − 0.31, *p* = 0.001), whereas the nonlinear effect of empathic disequilibrium was not significant (*b* = 0.11, 95% CI [− 0.21, 0.44], *β* = 0.12, *p* = 0.50). Additionally, both very high and very low levels of overall empathy were associated with increased eating disorder symptoms (*b* = 0.18, 95% CI [0.02, 0.33], *β* = 0.18, *p* = 0.03). However, a significant linear effect of overall empathy indicated that higher empathy was more strongly associated with symptom severity than lower empathy (*b* = 0.35, 95% CI [0.15, 0.54], *β* = 0.23, *p* = 0.001).

These effects appear to be largely driven by variation in AE (Table S1), although the polynomial model demonstrated superior fit to the data compared to simpler models (*χ*²(3) = 9.63, *p* = 0.02), suggesting that considering the intrapersonal relationship between CE and AE offers a more accurate representation of the data.

When controlling for anxiety and depressive symptoms, only the association between empathic disequilibrium toward AE dominance and eating disorder symptoms remained significant (*b* = − 0.30, 95% CI [− 0.56, − 0.03], *β* = −0.20, *p* = 0.03). As in the previous analysis, the nonlinear effect of empathic disequilibrium was not significant (*b* = 0.04, 95% CI [− 0.26, 0.34], *β* = 0.04, *p* = 0.79). Overall empathy was not associated with eating disorder symptoms, either linearly (*b* = 0.14, 95% CI [− 0.04, 0.33], *β* = 0.09, *p* = 0.13) or nonlinearly (*b* = 0.05, 95% CI [− 0.09, 0.20], *β* = 0.05, *p* = 0.48). Beyond the effects of the polynomial terms, both anxiety (*b* = 0.04, 95% CI [0.003, 0.07], *β* = 0.14, *p* = 0.04) and depressive symptoms (*b* = 0.14, 95% CI [0.09, 0.19], *β* = 0.33, *p* = 2 × 10^− 7^) were associated with eating disorder symptoms, however, sex was not significantly related to eating disorder symptoms in this model (*b* = 0.26, 95% CI [− 0.07, 0.59], *β* = 0.08, *p* = 0.13).

#### Interim discussion

The findings suggest that a tendency to feel others’ emotions more than to understand them, reflected in AE-dominance, is associated with eating disorder symptoms, even after accounting for depressive and anxiety symptoms. This supports the relevance of empathic disequilibrium in the context of eating disorders. However, as this is the first study to examine this relationship, replication in an independent sample is warranted. Furthermore, the relatively small number of participants with a clinical eating disorder diagnosis limited our ability to explore these associations within a diagnosed group, an important direction for future research. Finally, getting further insights into the underlying mechanisms underlying these associations is also important. As such, Study 2 used an independent sample to examine emotional reactivity as a potential mediator of the link between empathic disequilibrium and eating disorder symptoms as well as diagnosis.

## Study 2

### Methods

#### Participants

For this study, we recruited 835 participants. Participants were recruited through multiple means in the United Kingdom, including from the student recruitment system at Bournemouth University (*N* = 743), Prolific (*N* = 48), and leaflets (*N* = 44).

The mean age of participants was 25.79 years (*SD* = 11.71; range = 17–81). The majority identified as White (84.55%, *N* = 706). Other ethnic backgrounds included Asian (3.35%, *N* = 28), Black (including Black British and Black African; 2.16%, *N* = 18), Middle Eastern (including Turkish and Arab; *N* = 12), and mixed-race (predominantly mixed White and Black; *N* = 25). One participant identified as Latin American, and 45 participants (5.39%) preferred not to disclose their ethnicity. All participants disclosed their sex, with 80.36% (*N* = 671) assigned female at birth. Most participants identified as cisgender women (*N* = 635) or cisgender men (*N* = 150), 13 identified as transgender men, 12 as transgender women, 11 as non-binary, and 14 reported other gender identities (e.g., genderfluid, agender). The majority of participants identified as heterosexual (*N* = 573, 68.62%), followed by bisexual (*N* = 154, 18.44%), homosexual (*N* = 21, 2.51%), pansexual (*N* = 18), asexual (*N* = 7), queer (*N* = 6), and other (*N* = 3). Three participants preferred not to label their sexuality, and 50 preferred not to respond.

Most participants had indicated never having been diagnosed with a mental health condition (*N* = 475; 56.89%), while 292 (34.97%) reported a diagnosis. Sixty-eight participants (8.14%) preferred not to answer. Among those diagnosed, the most commonly reported conditions were anxiety disorders (*N* = 206), which included generalised anxiety disorder (*N* = 26), social anxiety (*N* = 8), panic disorder (*N* = 4), agoraphobia and specific phobias (*N* = 2). This was followed by diagnoses of depression (*N* = 174), PTSD or complex PTSD (*N* = 25), OCD (*N* = 21), personality disorders (*N* = 21; primarily borderline personality disorder), bipolar disorder (*N* = 7), psychotic spectrum disorders (*N* = 2), body dysmorphic disorder (*N* = 1), substance use disorder (*N* = 1), dissociative disorder (*N* = 1), and gender dysphoria (*N* = 1). Comorbidity was common, with 147 participants reporting multiple diagnoses, most frequently a combination of depression and anxiety (*N* = 97), or depression, anxiety, and additional conditions (*N* = 29). A small minority identified that they had been diagnosed as neurodivergent (ADHD: *N* = 13; autism: *N* = 37).

In total, 103 (12.34%) participants reported a diagnosis of an eating disorder (*N* = 63 with AN; *N* = 20 with BN; *N* = 5 with BED; *N* = 4 with other specified feeding and eating disorders; *N* = 7 with a combination of AN and BN; *N* = 1 with a restrictive eating disorder; and *N* = 4 unspecified or unsure). Of those diagnosed, 95 provided the date (or dates) of their diagnosis. At the time of the study, 33 participants reported an active eating disorder, 24 were in remission, and 38 were in partial remission or had recently experienced a relapse.

#### Procedure and measures

As in Study 1, participants were recruited online and, after providing informed consent, completed a battery of demographic and self-report measures. In this sample, McDonald’s ω for the IRI cognitive and affective empathy subscales were 0.82 and 0.76, respectively. Good reliability was also found for the EDE-Q (ω = 0.95).

In addition, participants completed the Emotional Reactivity Scale (ERS [46]). The ERS consists of 21 items rated on a scale from 0 to 4 and assesses three aspects of emotional reactivity: emotional sensitivity (how easily one becomes emotional), intensity, and persistence (how long emotional responses last). The ERS demonstrates strong construct validity with measures of behavioural inhibition and negative affect, and excellent criterion-related validity, successfully discriminating individuals with mood, anxiety, and eating disorders from healthy controls [46, 64]. For the current analyses, we used only the total score, which demonstrated excellent internal consistency (ω = 0.96).

#### Missing data handling

Little’s test indicated that missingness was not completely at random (*χ*²(10) = 49.9, *p* < 0.001). However, the overall proportion of missing data on key variables was low (4.43%) and generally considered negligible, suggesting minimal bias is expected [65]. To further reduce potential bias due to non-random missingness, as in Study 1, full information maximum likelihood was used [66]. Depressive and anxiety symptom measures, emotional reactivity (when not included as a model variable), and age were included as auxiliary variables. Table 3 presents the percentage of missing data for each key variable.

#### Statistical analysis

Similar to the first study, polynomial regression with response surface analysis was used to model overall empathy and empathic disequilibrium. Emotional and cognitive empathy scores were standardised prior to model inclusion.

We first examined the association between empathy constructs and eating disorder diagnosis by fitting a logistic polynomial regression model. Due to the small number of males in the diagnosed group (*N* = 4), sex was not included as a covariate to avoid biased estimation of odds ratios [67]. Given that the linear coefficient for empathic disequilibrium is bipolar (i.e., both positive and negative values reflect imbalance but in opposite directions), the inverse odds ratio (1/OR) was reported to aid interpretation of affective empathy dominance. Standard polynomial regression was conducted to examine the association between empathic disequilibrium, overall empathy, and symptoms of eating disorders, aiming to replicate the findings found in Study 1.

Mediation Analysis: To explore the potential mechanism underlying the association between empathic disequilibrium and eating disorder symptoms, we conducted a mediation analysis focusing on emotional reactivity as a possible mediator. This analysis followed methodological approaches outlined in our previous work [15, 16], along with best-practice recommendations for mediation analysis provided by [68].

Specifically, we first tested whether emotional reactivity was significantly associated with the response surface parameters (path A). We then examined whether emotional reactivity predicted eating disorder symptoms while controlling for the polynomial terms (path B). The indirect effect (path AB) was calculated as the product of paths A and B. To assess its significance, we computed 95% confidence intervals using Monte Carlo resampling with 10,000 iterations, implemented via the *MonteCarloCI* function from the *semTools* package (v0.5-6.941 [57]). An indirect effect was considered significant if the confidence interval did not include zero.

Sex was included as a covariate in all models. In addition, given prior evidence suggesting that emotional processes contributing to eating disorder symptoms may differ between males and females [29], we conducted multigroup analyses to examine whether the indirect effect via emotional reactivity was moderated by sex.

We are mindful of the limitations associated with conducting mediation analyses on cross-sectional data [69]. Therefore, to enhance the robustness of our findings, we incorporated several additional steps. First, we tested the alternative mediation model to evaluate the specificity of our proposed theoretical pathway, where empathic disequilibrium was treated as a mediator of the association between emotional reactivity and eating disorder symptoms. Given that polynomial response surface parameters cannot meaningfully serve as outcomes in such models [70], empathic disequilibrium was operationalised using traditional difference scores, specifically, the standardised score of cognitive empathy subtracted from affective empathy. Both mediation models (with either emotional reactivity or empathic disequilibrium as the mediator) were estimated following the same procedures described above. Total empathy, computed as the sum of standardised cognitive and affective empathy scores, was included as a covariate.

Second, we conducted a sensitivity analysis based on recommendations by Georgeson et al. [69], designed to simulate the inclusion of unmeasured variables and evaluate the robustness of the indirect effects as if longitudinal data had been collected within a cross-lagged panel model. This approach requires approximating the autoregressive (the stability of the mediator and outcome over time) and cross-lagged (the prediction of one variable on changes in another at a later time point, controlling for earlier measurement) correlations between the mediator and the outcome over time. To this end, we drew on prior research to inform a plausible range of autoregressive correlations for the ERS [71–73], and the EDE-Q [74–76]. Based on this literature, we selected autoregressive values ranging from 0.37 to 0.7, with 0.7 representing the upper bound permitted by the sensitivity analysis framework. Although no effect size estimation of longitudinal cross-lagged effects between emotional reactivity and eating disorder symptoms using the ERS and EDE-Q could be located, we used evidence from closely related variables [77, 78] to inform a more conservative cross-lagged correlation range of − 0.4 to 0.4, which is higher than reported values found. The analysis was implemented using the R code provided by Georgeson et al. [69].

#### Power analysis

The sample size was drawn from multiple convenience samples collected during and after data collection for the first study. However, parameters estimated from Study 1 allowed us to perform an independent and accurate power analysis for the current investigation. Using these parameters, we conducted a Monte Carlo–based power analysis to determine the smallest effect size detectable with our sample for the most stringent test in this study (the mediation effect in the multigroup analysis). Assuming a moderate association between empathic disequilibrium and emotional reactivity (see [15, 16]), 5000 Monte Carlo simulations indicated that our sample size was sufficient to detect a small indirect effect of 0.18 with a power (*1–β*) of 0.93 and *α* < 0.05. The analysis was conducted using the *simsem* package (v0.5-16 [63]).

### Results

Descriptive statistics are shown in Table 3, followed by a correlation matrix in Table 4. Participants who reported a diagnosis of an eating disorder had a mean EDE-Q score of 3.54 (*SD* = 1.54), which was markedly higher than the mean score among those without a diagnosis (*t*(831) = 9.77, *p* = 2 × 10^− 21^, *d* = 1.03).

**Table 3:** Descriptive statistics of Study 2

Variable	Mean (SD)	% Missing data
IRI—cognitive empathy	34.17 (8.54)	0.12
IRI—affective empathy	33.01 (7.13)	0.24
EDE-Q	2.19 (1.57)	0.24
ERS	41.19 (22.23)	6.95%

*IRI* Interpersonal Reactivity Index, *EDE-Q* Eating Disorder Examination Questionnaire, *ERS* Emotional Reactivity Scale

**Table 4:** Correlation matrix

Variable	1	2	3
1. Cognitive empathy			
2. Affective empathy	0.45**		
3. EDE-Q	0.01	0.14**	
6. ERS	0.08*	0.38**	0.42**

*EDE-Q* Eating Disorder Examination Questionnaire, *ERS* Emotional Reactivity Scale. * *p* < 0.05. ** *p* < 0.01

#### Eating disorder diagnosis

Before exploring the results, the logistic regression assumptions were assessed. Cook’s distance was calculated using the *broom* package (v1.0.6 [79]) to assess the influence of potential outliers. It revealed that no participant exceeded the 0.5 threshold, with the highest Cook’s distance being 0.10. The highest VIF observed was 2.70, indicating no major multicollinearity issues. Additionally, the Box-Tidwell test, conducted with the *car* package (v3.1-2 [80]), confirmed the linearity assumption between predictors and the log odds of diagnosis, with all *p*-values above 0.1. Collectively, these results suggest that the assumptions of logistic regression were adequately met.

**Fig. 2:**
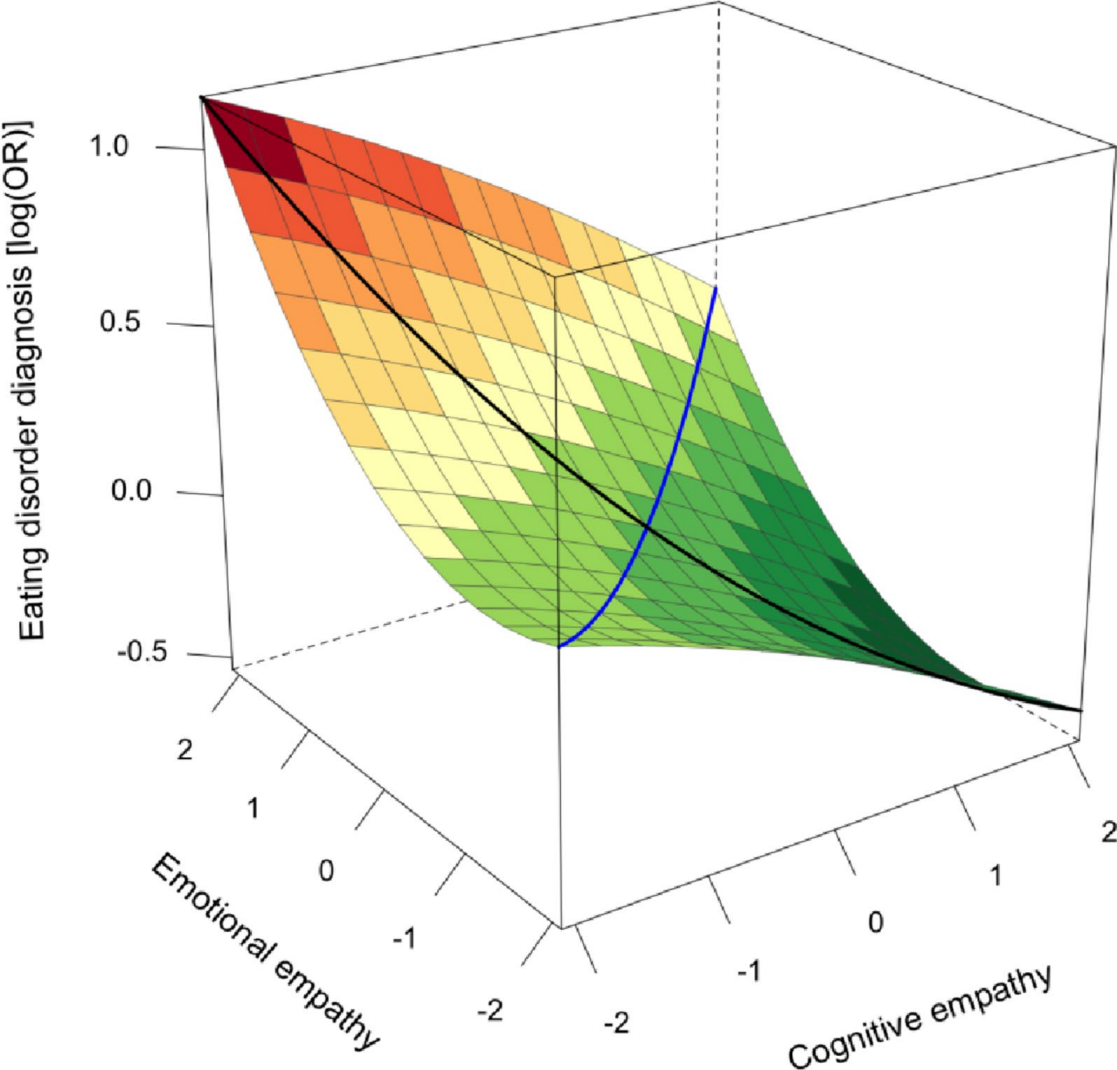
Logistic polynomial regression with response surface analysis predicting eating disorder diagnosis. The black line represents empathic disequilibrium, with movement toward the left corner indicating AE dominance and movement toward the right corner indicating CE dominance. The blue line represents overall empathy, with movement along this line reflecting increasing levels of combined AE and CE. Log-odds probability of eating disorder diagnosis is represented by the colour gradient, with cooler colours (dark green) indicating lower symptom severity and warmer colours (yellow to red) indicating higher symptom severity

The polynomial regression model accounted for 4.9% of the variance in eating disorder diagnosis, and the polynomial surface is depicted in Fig. 2. Including the polynomial terms significantly improved model fit (*χ*²(3) = 74.44, *p* < 5 × 10⁻¹⁶). Among the surface parameters, only empathic disequilibrium toward AE dominance significantly predicted diagnosis (1/OR = 1.46, 95% CI [1.17, 1.82], *p* = 0.001). No non-linear association with empathic disequilibrium was found (OR = 1.08, 95% CI [0.83, 1.41], *p* = 0.56). Furthermore, overall empathy showed no significant relationship with eating disorder diagnosis either linearly (OR = 1.01, 95% CI [0.88, 1.15], *p* = 0.92) or non-linearly (OR = 1.06, 95% CI [0.96, 1.17], *p* = 0.28).

#### Eating disorder symptoms

The residuals of the model showed a mildly positively skewed distribution (skew = 0.41, kurtosis = − 0.96). Attempts to correct this deviation from normality, such as applying square root or logarithmic transformations to the positively skewed EDE-Q scores, did not substantially improve the residual distribution. Given that the deviation from normality is considered mild [81] and to maintain interpretability, the analysis proceeded without transforming the data. VIFs were all below 2.20, indicating no significant multicollinearity among predictors.

**Fig. 3:**
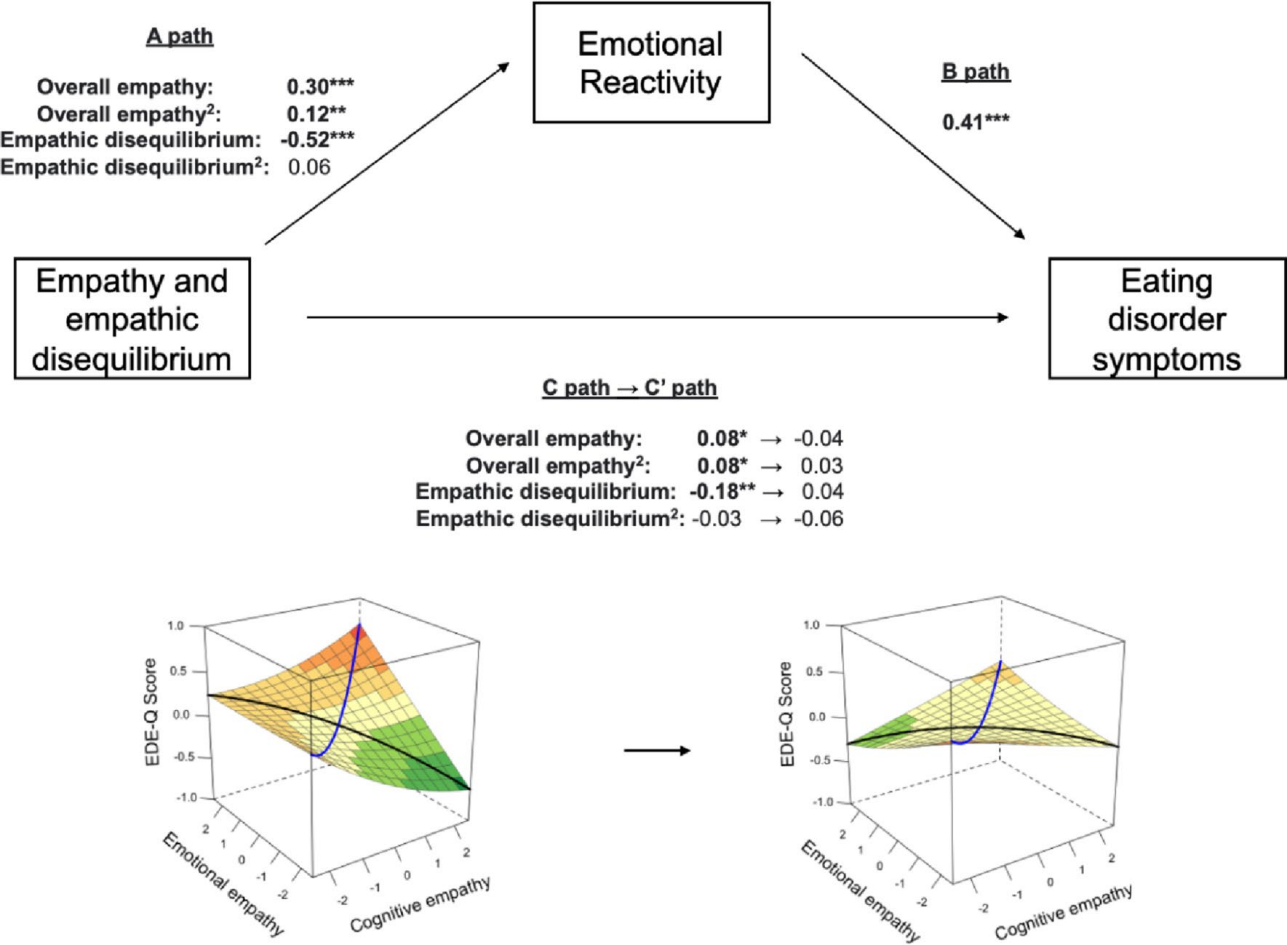
Mediation model. The plots depict the polynomial surface illustrating the relationship between empathic disequilibrium and eating disorder symptoms before (left) and after (right) controlling for emotional reactivity. Eating disorder symptom severity is represented by the colour gradient, with cooler colours (dark green) indicating lower symptom severity and warmer colours (yellow to red) indicating higher symptom severity. To enable comparability between the two plots, EDE-Q scores were standardised, and the plot dimensions were scaled to display one standard deviation above and below the mean. * *p* < 0.05, ** *p* < 0.01, *** *p* < 0.001

Similar to the results in Study 1, the polynomial regression model explained 5.4% of the variance in eating disorder symptoms and showed an improved fit compared to a model including only CE and AE (*χ*²(3) = 7.61, *p* = 0.05). No differences in surface parameters between males and females (*χ*²(4) = 5.15, *p* = 0.27) were found. Although effect sizes were smaller than in Study 1, the results replicated the key finding that empathic disequilibrium towards AE dominance was associated with greater eating disorder severity (*b* = − 0.28, 95% CI [− 0.48, − 0.07], *β* = − 0.18, *p* = 0.01), with no evidence of a non-linear relationship (*b* = − 0.05, 95% CI [− 0.29, 0.20], *β* = − 0.03, *p* = 0.71). Both linear (*b* = 0.13, 95% CI [0.003, 0.25], *β* = 0.08, *p* = 0.05) and non-linear (*b* = 0.10, 95% CI [0.003, 0.19], *β* = 0.08, *p* = 0.04) associations with overall empathy were observed, primarily driven by higher levels of overall empathy (see left plot in Fig. 3).

#### Mediation analysis

Next, we tested whether the associations between empathy, empathic disequilibrium, and eating disorder symptoms were mediated by emotional reactivity (Fig. 3). The models did not significantly differ between males and females (*χ*²(11) = 13.56, *p* = 0.26). Although the model residuals showed mild skewness and kurtosis (skew = 0.36, kurtosis = − 0.96), transformations of the EDE-Q or ERS scores did not improve the distribution of residuals.

As hypothesised (and depicted in Figure S1), empathic disequilibrium toward AE dominance was associated with greater emotional reactivity (*b* = − 11.77, 95% CI [− 14.62, − 8.92], *β* = − 0.52, *p* = 2 × 10^− 16^), with no evidence of a non-linear association (*b* = 0.86, 95% CI [− 2.48, 4.20], *β* = 0.06, *p* = 0.62). Overall empathy was positively associated with emotional reactivity, both linearly (*b* = 6.73, 95% CI [5.04, 8.42], *β* = 0.30, *p* = 5 × 10^− 15^) and non-linearly (*b* = 1.86, 95% CI [0.57, 3.14], *β* = 0.12, *p* = 0.01). In turn, emotional reactivity was positively associated with eating disorder symptoms (*b* = 0.029, 95% CI [0.024, 0.034], *β* = 0.41, *p* = 5 × 10^− 30^).

In line with our hypothesis, emotional reactivity mediated the association between AE dominance and eating disorder symptoms (*b* = − 0.33, 95% CI [− 0.44, − 0.24], *β* = − 0.21). It also mediated the linear (*b* = 0.19, 95% CI [0.14, 0.26], *β* = 0.12) and non-linear (*b* = 0.05, 95% CI [0.02, 0.09], *β* = 0.05) associations with overall empathy. The mediation model is shown in Fig. 3.

Testing the model using the traditional difference scores, a significant indirect effect was observed (*b* = − 0.19, 95% CI [− 0.25, − 0.14], *β* = − 0.13), consistent with the findings from the polynomial regression with response surface analysis. In line with our theoretical model, the alternative mediation model, with empathic disequilibrium as the mediator of the association between emotional reactivity and eating disorder symptoms, was not supported (*b* = − 0.00001, 95% CI [− 0.002, 0.001], *β* = − 0.0002).

Furthermore, sensitivity analyses suggested that, under plausible assumptions from the literature, the indirect effect in a longitudinal design could range between − 0.31 and − 0.01, indicating the potential for a significant (*p* < 0.05) small to small-to-moderate effect size (Figure S2).

## Discussion

The present study examined the role of empathic disequilibrium—the imbalance between cognitive and affective empathy (CE and AE respectively)—in eating psychopathology across two independent samples comprising 345 undergraduate students (Study 1) and 835 undergraduates and community participants, including 103 individuals with eating disorder diagnoses (Study 2). Our findings provide novel evidence that empathic disequilibrium characterised by AE-dominance is consistently associated with both eating disorder diagnosis and symptom severity across both clinical (assessed via self-report) and non-clinical populations. Furthermore, we identified emotional reactivity as a key mediating mechanism through which this empathic imbalance contributes to eating disorder pathology, with the mediation pathway accounting for the full relationship between empathic disequilibrium and eating disorder symptoms. These results advance our understanding of emotional processes in an interpersonal context in eating disorders and suggest that examining the relative balance between empathic capacities may be more informative than considering CE and AE in isolation.

Our findings extend previous research on empathy in eating disorders by demonstrating that it is *disequilibrium* in the balance between CE and AE, rather than deficits in specific empathic components, that seems associated with eating disorder pathology. Kerr-Gaffney et al.‘s [6] comprehensive meta-analysis of 17 studies found that eating disorders are characterised by selective CE deficits with preserved AE. However, our results suggest a different interpretation of these patterns, as we found that it is the *relative dominance* of AE that drives eating disorder pathology: that is, when AE is disproportionately high relative to CE within individuals. Importantly, CE itself was entirely unrelated to symptoms of eating disorder. This interpretation reconciles several inconsistencies in the literature that have puzzled researchers. For instance, whilst most studies have shown that people with AN tend to have lower levels of CE (see [5, 6]), some studies report higher [82, 83], lower [84], or no significant differences in personal distress [85–87]. The empathic disequilibrium framework described herein and in previous work [12–17, 27, 28] suggests that these seemingly contradictory findings may reflect the fact that it is not only the absolute level of CE or AE, but also their relative strength compared to the other, that matters clinically.

Another significant finding is the identification of emotional reactivity as a potential mediating mechanism, which provides important insights into the processes through which empathic disequilibrium contributes to eating disorder pathology. The robustness of our mediation pathway was further supported by our sensitivity analyses, which indicated that the indirect effect would likely remain significant in longitudinal designs. Interestingly, most of the literature so far has looked at emotional reactivity in an intrapersonal context (e.g. [35]), but our study suggests that interpersonal contexts may also be involved in explaining this heightened emotional reaction. Overall, our findings suggest that an AE-dominance experience may lead to hyper-arousal when encountering others’ emotions, requiring emotion regulation strategies to manage it. While AE-dominance and emotional reactivity were previously linked to NSSI as another means of emotion regulation [16], the present data suggests they may also lead to maladaptive disordered eating behaviours as a coping mechanism, in line with the known role that eating disorder behaviours play in emotion regulation [1–3, 29, 31, 35, 88, 89]. The cognitive interpersonal model for AN developed by Treasure and Schmidt [7, 90] explain that problems in social cognition and difficulties with interpersonal relationships can predispose individuals to developing AN. Whilst the model and literature explain that adverse social experiences, such as childhood maltreatment, insecure attachment, or social exclusion, can shape these interpersonal relationship difficulties, our results suggest that empathic disequilibrium may also be at play.

### Limitations

Several limitations should be acknowledged when interpreting these findings, though many point toward important avenues for future research. First, the cross-sectional nature of our data precludes definitive causal inferences about the temporal relationships between empathic disequilibrium, emotional reactivity, and eating disorder symptoms. Despite our comprehensive sensitivity analyses, prospective research is essential to establish whether empathic disequilibrium precedes eating disorder symptomatology or emerges as a consequence of the disorder. Longitudinal studies could also examine whether empathic disequilibrium represents a stable (i.e. trait) vulnerability factor or whether it fluctuates with symptom severity and treatment response. Whilst there is limited literature comparing empathy in acute and remission phases of AN, a recent systematic review found that difficulties with empathy may show some improvement following recovery, though evidence remains inconsistent [5]. As such, investigating the stability of empathic disequilibrium across different stages of illness and recovery in future studies would be particularly valuable.

Second, although the use of self-reported diagnoses represents a limitation, existing evidence supports their validity in psychiatric research [91], and this approach allows for the efficient recruitment of larger samples. Importantly, whilst our sample included individuals with various eating disorder diagnoses, including a comparatively higher number of participants with binge-eating features than is typically represented in the literature, there was an overrepresentation of AN cases. We did not have enough power to run separate analyses for each eating disorder type, which potentially limits generalisability to all eating disorder presentations. This is particularly important given that the cognitive interpersonal model [7, 90] was developed for AN, and given the emerging evidence that empathy profiles may differ across eating disorder subtypes [6, but see 39 and 40].

Third, the generalisability of our samples was also limited by lack of diversity relating to sex, gender and ethnicity. Our samples predominantly comprised people assigned female at birth who also identified as women: whilst this reflects the higher prevalence of eating disorders in women, it limits our ability to examine potential sex and gender differences in empathic disequilibrium patterns. Our models were not moderated by sex, but past research has shown sex differences in empathic processing in the general population [92], so we recommend that future research should specifically examine whether empathic disequilibrium operates similarly across sexes, genders and eating disorder subtypes. Our sample also predominantly comprised White university students, which limits the generalisability of our findings to broader and more culturally diverse populations. To address this limitation, we recommend that future research replicate these findings in samples that better reflect the cultural diversity of the wider community. In particular, greater attention should be given to factors such as ethnic identity, acculturation, and acculturative stress [93], as these processes can shape how individuals navigate emotional experiences and interpersonal dynamics.

Fourth, whilst we controlled for key demographic variables and comorbid depression and anxiety, unmeasured confounding variables could potentially explain our findings. For instance, alexithymia—the difficulty identifying and describing one’s own emotions—is elevated in eating disorders [30] and could confound relationships between empathic functioning and emotional reactivity. Our reliance on self-report measures brings in additional bias, as difficulties identifying their feelings could extend to their perception of their own empathic abilities. Incorporating behavioural or physiological measures of empathy would strengthen future investigations and provide convergent validity for self-report findings.

### Implications

The clinical implications of our findings are substantial and suggest several potential avenues for enhancing eating disorder treatment approaches. Current evidence-based treatments for eating disorders primarily focus on intrapersonal processes, including nutritional rehabilitation and cognitive-behavioural interventions targeting eating disorder cognitions [94]. However, our results suggest that interpersonal emotional processes—specifically empathic functioning—may also warrant therapeutic attention. Therapeutic interventions addressing empathic disequilibrium as well as emotional reactivity might focus on helping reduce distress, which would align with a Dialectical Behaviour Therapy (DBT) approach. DBT has shown efficacy in eating disorders [95], specifically in targeting emotional reactivity through distress tolerance and emotion regulation skills. Our paper suggests that discussing interpersonal emotional difficulties to improve affect regulation during social exchanges may also prove helpful. Family-based treatments and interpersonal therapies for eating disorders could also be informed by our findings. For example, understanding that individuals with eating disorders may experience emotional hyper-arousal in response to others’ emotions could help family members and therapists adjust their communication styles and emotional expressions during treatment. Being aware of the imbalance could also help therapists adapt their support to help create balance by either focusing on understanding others better or on regulating one’s emotional responses to others’ feelings, or both. In addition, increasing awareness of empathic disequilibrium may enhance self-understanding, for instance by helping individuals recognise the social contexts that trigger their emotional responses [13]. As such, we suggest that incorporating the concept of empathic disequilibrium into psychoeducational approaches for eating disorders could further support emotional insight and resilience in social situations.

## Conclusion

In conclusion, this study provides the first comprehensive evidence that empathic disequilibrium characterised by affective empathy dominance represents a potential risk factor for eating psychopathology, operating through heightened emotional reactivity. Our findings demonstrate that the *balance* between empathic capacities is more predictive than individual components, challenging traditional approaches to understanding empathy in psychopathology and highlighting the importance of considering interpersonal emotional processes in eating disorder conceptualisation and treatment. The consistency of effects across two independent samples and both clinical (assessed via self-report) and non-clinical populations strengthens confidence in the clinical relevance of these results, although longitudinal studies replicating our findings are warranted. These findings advance our understanding of eating disorders by identifying a previously overlooked interpersonal pathway to symptom development and maintenance, opening new avenues for research and intervention that address both intrapersonal and interpersonal dimensions of emotional functioning to improve outcomes for affected individuals.

## Supplementary Information

Below is the link to the electronic supplementary material.

Supplementary Material 1.

## Data Availability

The datasets used and/or analysed during the current study are available from the corresponding author on reasonable request.

## Abbreviations

AE: Affective empathy
AN: Anorexia nervosa
BED: Binge Eating Disorder
BN: Bulimia nervosa
CE: Cognitive empathy
CES-D: Centre for Epidemiologic Studies Depression Scale
DBT: Dialectical behavioural therapy
ED: Eating disorder
EDEQ: Eating Disorder Examination Questionnaire
ERS: Emotional Reactivity Scale
GAD-7: Generalised Anxiety Disorder scale
OR: Odd ratio
