# Supplementary material for: Feeling more than understanding: empathic disequilibrium and emotional reactivity in eating psychopathology
Source: J Eat Disord. 2026 Apr 18;14:135. doi: 10.1186/s40337-026-01600-2 (PMC13267261; doi:10.1186/s40337-026-01600-2)
Supplement: Supplementary file 1 — Supplementary Material 1. [file 40337_2026_1600_MOESM1_ESM.docx]

**Supplementary Information**

**Table S1**

***Polynomial regression coefficients for all models***

|  | **Model** | **CE** | **EE** | **CE²** | **CE × EE** | **EE²** |
| --- | --- | --- | --- | --- | --- | --- |
| **Study 1** | **EDE-Q** | –0.06 [–0.23, 0.11] | 0.41 [0.23, 0.59] *** | 0.10 [–0.04, 0.23] | 0.03 [–0.15, 0.22] | 0.05 [–0.06, 0.16] |
|  | **EDE-Q with covariates** | –0.08 [–0.23, 0.07] | 0.22 [0.05, 0.39] ** | 0.03 [–0.09, 0.15] | 0.01 [–0.16, 0.17] | 0.02 [–0.08, 0.12] |
|  | **CES-D** | 0.09 [–0.31, 0.50] | 0.84 [0.41, 1.28] *** | 0.39 [0.06, 0.71] * | 0.04 [–0.41, 0.48] | 0.15 [–0.11, 0.41] |
|  | **GAD-7** | 0.08 [–0.54, 0.69] | 1.93 [1.27, 2.59] *** | 0.39 [–0.10, 0.88] | 0.56 [–0.12, 1.23] | 0.25 [–0.15, 0.64] |
| **Study 2** | **Eating disorder diagnosis†** | 0.83 [0.73, 0.95]** | 1.21 [1.07, 1.37]** | 0.99 [0.88, 1.10] | 0.99 [0.86, 1.13] | 1.08 [1.00, 1.18] |
|  | **EDE-Q** | –0.07 [–0.19, 0.04] | 0.20 [0.08, 0.32] ** | 0.03 [–0.07, 0.12] | 0.07 [–0.06, 0.20] | 0.001 [–0.09, 0.09] |
|  | **EDE-Q with ERS** | –0.00 [–0.11, 0.11] | –0.06 [–0.18, 0.06] | 0.00 [–0.09, 0.09] | 0.06 [–0.06, 0.18] | –0.02 [–0.10, 0.07] |
|  | **ERS** | –2.42 [–4.03, –0.82] ** | 9.15 [7.52, 10.79] *** | 0.79 [–0.51, 2.09] | 0.50 [–1.26, 2.25] | 0.57 [–0.63, 1.76] |

*Note*. * *p* < 0.05, ** *p* < 0.01, *** *p* < 0.001; ^†^ reported in OR

**Figure S1**

***Polynomial response surface predicting emotional reactivity***
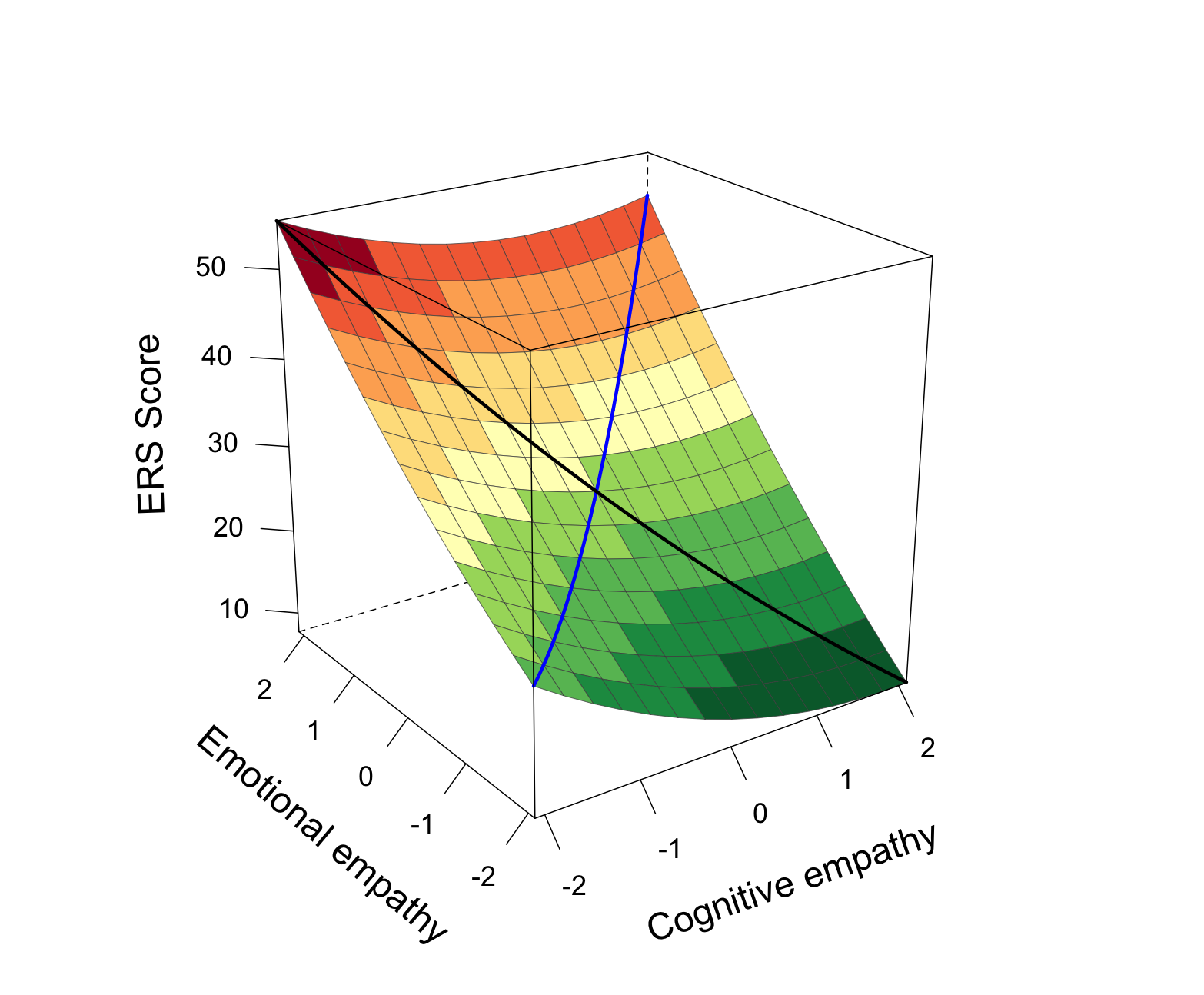


*Note*. The black line represents empathic disequilibrium, with movement toward the left corner indicating emotional empathy dominance and movement toward the right corner indicating cognitive empathy dominance. The blue line represents overall empathy, with movement along this line reflecting increasing levels of combined emotional and cognitive empathy. Emotional reactivity is colour-coded for interpretability.

**Figure S2**

***Sensitivity analysis plot for the indirect effect by autoregressive and cross-lagged correlations***

**
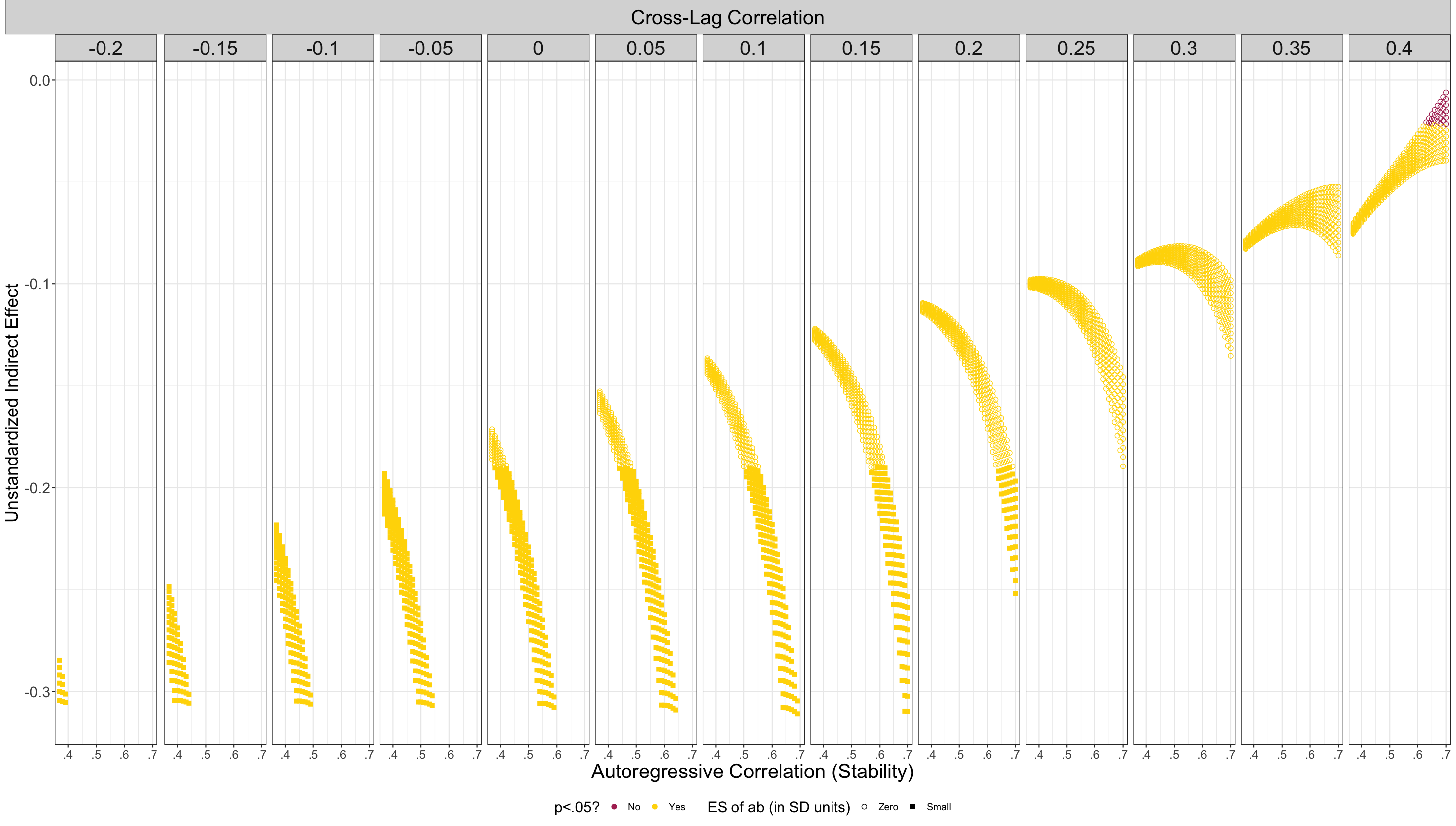
**

*Note*. Sensitivity analysis for the indirect effect from a cross-sectional mediation model, based on the approach of Georgeson et al. (2023). Indirect effect estimates (vertical axis) are shown across a grid of possible autoregressive correlations (horizontal axis) and cross-lag correlations (facets) reflecting hypothesised longitudinal stability and temporal associations. Each point represents the indirect effect computed under a given parameter combination; yellow indicates p < 0.05 and purple indicates p > 0.05.
